# Multiple spinal brown tumors presenting as compressive thoracic myelopathy in a patient on maintenance hemodialysis

**DOI:** 10.1093/jscr/rjaf821

**Published:** 2025-10-17

**Authors:** Sharvari Joshi, Ravindra A Prabhu, Indu R Rao

**Affiliations:** Department of Medicine, Kasturba Medical College, Tiger Circle Road, Madhav Nagar, Manipal 576104, India; Department of Nephrology, Kasturba Medical College, Tiger Circle Road, Madhav Nagar, Manipal 576104, India; Department of Nephrology, Kasturba Medical College, Tiger Circle Road, Madhav Nagar, Manipal 576104, India

**Keywords:** secondary hyperparathyroidism, brown tumor, surgical intervention

## Abstract

Brown tumor, also known as osteitis fibrosa cystica, is a benign bone lesion due to excessive lytic action by osteoclasts, in the presence of primary, secondary, or tertiary hyperparathyroidism. Due to early screening, they are a rare occurrence. This report presents a case of a 47-year-old man on regular hemodialysis presenting with compressive thoracic myelopathy. Based on elevated parathyroid hormone, imaging, and histopathology, the diagnosis of brown tumor was confirmed. He then underwent tumor excision, following which the underlying secondary hyperparathyroidism was treated with calcitriol and calcium replacement. Though secondary hyperparathyroidism is a common complication of chronic kidney disease, brown tumors are a rare manifestation and uncommonly located in the spine. Differential diagnoses include metastatic lesions, plasmacytomas, giant cell granulomas, or simple bone cysts. Early diagnosis and management of a spinal brown tumor can prevent neurological complications.

## Introduction

Brown tumors are rare nonneoplastic lytic bony lesions resulting from hyperparathyroidism [1–3]. Secondary hyperparathyroidism is a common complication of chronic kidney disease [3, 4]. Reduced production of calcitriol and reduced excretion of phosphorus raise the parathyroid hormone (PTH) levels [3, 4]. Prolonged elevation of PTH levels may lead to increased focal osteoclastic activity, bone resorption, and proliferation of fibroblasts, leading to the formation of Brown tumors [3, 4]. Brown tumors are more commonly seen in the maxilla and mandible, followed by other facial bones, pelvis, ribs, femurs, and other long bones, and less commonly in the axial skeleton [2]. In this report, we discuss a case of a 47-year-old gentleman presenting with compressive thoracic myelopathy secondary to a Brown tumor, undergoing hemodialysis.

## Case report

A 47-year-old man presented with progressive upper back pain for 1 month, which was insidious in onset, progressive, radiating to the proximal thigh, and aggravated on bending forwards. He did not report any motor or sensory symptoms, bowel or bladder disturbance, or constitutional symptoms. History was significant for hypertension and chronic kidney disease, having been diagnosed with Immunoglobin A (IgA) nephropathy 15 years ago, status-post two failed kidney transplants—the first from 2010 to 2014, and the second from 2018 to 2020—and has been on maintenance hemodialysis since 2020. Examination of the spine revealed a mild kyphotic deformity and tenderness over the thoracic region. Neurological examination demonstrated paraparesis with muscle strength graded at 4/5, diminished sensation (1/2) in the L5 and S1 dermatomes, bilaterally brisk knee jerk reflexes, and a positive Babinski sign bilaterally.

**Figure 1:**
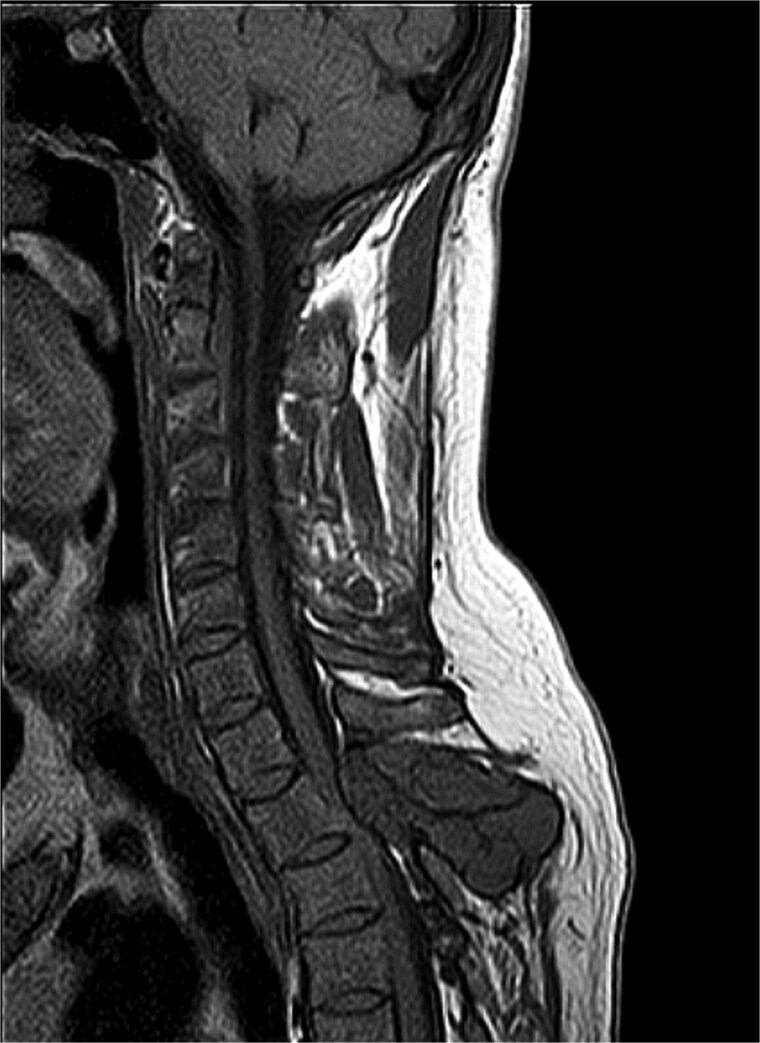
MRI thoracic spine: Extradural lobulated minimally enhancing lesions involving spinous process of T2 vertebra, right pedicle and lamina of T3 vertebra and posterior aspect of left T2 rib.

Magnetic resonance imaging (MRI) of the thoracic spine showed two expansile extradural lobulated lesions, the first of which involved the spinous process of the T2 vertebra with few hypointense non-enhancing septations within, anteriorly extending into the epidural space with narrowing of the spinal canal causing compression of the spinal cord with myelomalacic changes. A similar expansile lesion involving the right pedicle and lamina of the T3 vertebra and posterior aspect of the left T2 rib (Fig. 1). Parathyroid hormone was elevated at 713.6 pg/mL, Calcium was low at 2 mmol/L, phosphorus within normal limits at 1.10 mmol/L, while Alkaline phosphatase (ALP) was also elevated at 181 U/L (Table 1). Histopathological examination revealed spindle-shaped fibroblasts admixed with giant-cell-like osteoclasts, with areas of haemorrhage, and intracellular and stromal hemosiderin deposits. There were no mitoses or nuclear polymorphisms. A Tc-99 m sestamibi single-photon emission computed tomography/computed tomography (SPECT/CT) revealed soft tissue lesions inferior to lower poles of both thyroid lobes, the largest being 0.9 × 0.7 cm, suggestive of bilateral inferior parathyroid hyperplasia, without significant radiotracer uptake. Absence of significant tracer uptake is consistent with hyperplasia, where the glands are typically smaller and less rich in mitochondria-containing oxyphil cells as compared to adenomas [5].

**Table 1:** Laboratory investigation.

	At Presentation	4-month follow-up	6-month follow-up
PTH (pg/mL)	713.6	250	108
Serum Calcium (mmol/L)	2	2.1	2.2
Serum Phosphorous (mmol/L)	1.10	1.61	1.58
ALP (U/L)	181	84	97

In the context of secondary hyperparathyroidism with elevated PTH and characteristic histopathological findings, a diagnosis of brown tumor was established. Given the progressive nature of the symptoms, surgical intervention was decided upon in the form of tumor excision, decompression, and posterior spinal stabilization. Following surgery, the patient improved symptomatically and was initiated postoperatively on calcitriol 250 mcg three times a day and Calcium carbonate 1000 mg TID to maintain calcium homeostasis and control secondary hyperparathyroidism [1–4, 6]. Subsequently on follow-up after 4 months, PTH was found to be at 250 pg/mL. As per KDIGO (Kidney Disease: Improving Global Outcomes) recommendations for target PTH level in persons with end-stage renal disease, the aim is to maintain PTH levels in the range of ~2–9 times the upper normal limit, thus the PTH level in this patient was acceptable [6]. Ca was at 2.1 mmol/L, while Phosporus was at 1.61 mmol/L, and ALP was at 84 U/L. At the 6-month mark, PTH had improved further to 108 pg/mL, Ca was within limits at 2.2 mmol/L, phosphorus at 1.58 mmol/L, and ALP was 97 U/L (Table 1). There was no recurrence of symptoms on follow-up.

## Discussion

In secondary hyperparathyroidism, which commonly results from chronic kidney disease, low calcium and vitamin D stimulate the parathyroid glands to secrete an excessive amount of PTH [1–6]. Brown tumors are rare manifestations of hyperparathyroidism focal osteoclastic resorption accompanied by proliferation of granulation and fibrous tissue, as well as areas of hemorrhage and hemosiderin deposits [1–3, 6]. They usually develop in persons with very high PTH levels, often exceeding ten times the upper limit of normal, over a prolonged period [6].

Although often slow-growing and sometimes asymptomatic, brown tumors may present with symptoms depending on their location, including swelling, pain, pressure effects, or pathological fractures [1–3, 6]. Brown tumors located in the spine may present with back pain, motor or sensory symptoms, or bladder/bowel disturbances [1, 6]. They have nonspecific laboratory or imaging findings. On imaging, the lesions can mimic lesions such as metastases, myelomas, giant cell granulomas, or simple bone cysts [1]. Histopathologically, they demonstrate hemosiderin deposits, osteoclastic giant cells, and vascularized fibrous tissue, which can resemble other lesions such as giant cell tumors or aneurysmal bone cysts [1–3, 6]. Therefore, a combination of laboratory investigations, imaging, and biopsy, alongside a thorough history and physical examination, is essential for diagnosis.

Treatment involves managing the underlying hyperparathyroidism, which may lead to regression and resolution of the lesion [1–3, 6]. This includes aggressively lowering PTH by addressing hyperphosphatemia, hypocalcemia, and vitamin D deficiency [1–4, 6]. Parathyroidectomy may also result in lesion resolution. However, in symptomatic or refractory cases, surgical intervention may be necessary. In particular, lesions causing prolonged spinal cord compression require urgent surgery to prevent irreversible neurological damage [1–3, 7].

Previously, brown tumors occurred more commonly as a result of primary hyperparathyroidism, however, cases arising from secondary hyperparathyroidism are increasing due to longer life expectancy in patients with chronic kidney disease [1]. Additionally, brown tumors are often misdiagnosed as they can mimic various other bony lesions [1–3, 6]. Thus, it is important to consider brown tumors as a differential diagnosis, confirmed through a combination of clinical, laboratory, imaging, and histopathological findings. Moreover, spinal involvement is uncommon, warranting a high index of suspicion in patients with chronic kidney disease presenting with vertebral lesions to enable early diagnosis [1, 6]. This case highlights that early surgical excision, in addition to controlling hyperparathyroidism, may be required to prevent irreversible neurological damage from spinal cord compression [1, 6, 7].

## References

[1] Ramachandraiah , Kumar M, Kishen, et al. Brown tumor causing thoracic compressive myelopathy: a case report and review of literature. Indian Spine J 2021;4:203–13. 10.4103/isj.isj_48_20

[2] Fazaa A, Makhlouf Y, Miladi S, et al. Hyperparathyroidism: unusual location of brown tumors. Clin Case Rep 2022;10:e05376. 10.1002/ccr3.5376 35140968 PMC8813670

[3] Guedes A, Becker RG, Nakagawa SA, et al. Update on brown tumor of hyperparathyroidism. *Rev Assoc Med Bras* (1992) 2024;70:e2024S132. 10.1590/1806-9282.2024S132 38865551 PMC11164281

[4] London G, Coyne D, Hruska K, et al. The new kidney disease: improving global outcomes (KDIGO) guidelines - expert clinical focus on bone and vascular calcification. Clin Nephrol 2010;74:423–32. 21084045 PMC3770279

[5] Stephen AE, Roth SI, Fardo DW, et al. Predictors of an accurate preoperative sestamibi scan for single-gland parathyroid adenomas. Arch Surg 2007;142:381–6. 10.1001/archsurg.142.4.381 17441292

[6] Wiederkehr M . Brown tumor complicating end-stage kidney disease. Clin Nephrol Case Stud 2020;2020:72–9. 10.5414/CNCS110195 PMC7552353 33062583

[7] Robson P . Metastatic spinal cord compression: a rare but important complication of cancer. Clin Med (Lond) 2014;14:542–5. 10.7861/clinmedicine.14-5-542 25301920 PMC4951968

